# Operationalization of the social cognitive theory to explain and predict physical activity in Germany: a scale development

**DOI:** 10.3389/fspor.2024.1508602

**Published:** 2024-11-26

**Authors:** Viktoria S. Egele, Robin Stark

**Affiliations:** Department of Education, Saarland University, Saarbrücken, Germany

**Keywords:** scale, self-efficacy, measurement, assessment, social cognitive theory, physical activity

## Abstract

**Introduction:**

Social cognitive theory is one of the most prominent psychological theories regarding human behavior. Previous research tested and confirmed parts of the theory concerning the explanatory and predictive value of the theory, both in specific populations and in selected domains of physical activity. However, the value of this research is limited as researchers often use their own item sets rather than validated scales. Therefore, comparability of the studies is restricted and the quality of the individual findings can often not be conclusively assessed as psychometric properties of the measurement are unclear. The goal of this research was to develop a parsimonious, reliable, and valid questionnaire to assess the elements of SCT in the context of physical activity.

**Methods:**

In total, 90 items were developed for the four factors of SCT, which were then examined by exploratory factor analysis and reduced to 18 items in total.

**Results:**

Cross-validation was successful. Internal consistency was good for the four subscales, test-retest reliability was satisfactory, as were indicators for convergent and divergent validity.

**Discussion:**

A short, reliable, and valid instrument was developed intended for use in the general adult population in Germany for research on theoretical assumptions and interventions based on social cognitive theory.

## 1 Introduction

Physical activity is an important contributor to health and insufficient levels of physical activity have been shown to be related to noncommunicable diseases, coronary heart disease, cancer, poor mental health, and premature death (1, 2). A recent long-term study involving data from nearly two million people in 168 countries found that 27.5 percent of adults worldwide do not meet recommended levels of physical activity and must be classified as physically inactive (3). In Germany, rates of physical activity were found to be even lower, with 40%–49.9% of the population being classified as physically inactive (3).

Promoting physical activity is, therefore, a major goal of the World Health Organization (2), and an understanding of the factors that influence physical activity behavior has never been more important. Albert Bandura's social cognitive theory (SCT) is one of the most prominent psychological theories regarding the explanation of human behavior (4) and a recent synthesis of the literature indicated that the social cognitive framework remains the dominant approach to explain, predict, and change physical activity behavior (5–7). SCT assumes that personal, behavioral, and environmental factors interact to influence a persońs behavior – and thus enables a holistic analysis of social-cognitive factors theoretically associated with behavior. In the following, a brief overview of the constructs constituting social cognitive theory is given.

According to Bandura, self-efficacy is defined as a person's belief that he or she is capable of successfully accomplishing certain tasks. It is the belief that one has the necessary skills, resources, and strategies to achieve a certain goal. Multiple similar definitions stem from Bandura himself: “Perceived self-efficacy is defined as people's beliefs about their capabilities to produce designated levels of performance that exercise influence over events that affect their lives.” (8), (p. 71). Similar definitions have been given by Bandura in earlier and later works, for example, “Perceived self-efficacy is concerned with people's beliefs in their capabilities to produce given attainments” (9), (p. 1) and “Perceived self-efficacy is concerned with judgments of how well one can execute courses of action required to deal with prospective situations” (10), (p. 122). As such, “perceived self-efficacy is not a measure of the skills one has but a belief about what one can do under different sets of conditions with whatever skills one possesses” (10), (p. 37). Self-efficacy has been shown to be the strongest predictor of physical activity behavior, and numerous studies demonstrated both a direct and an indirect impact on behavioral outcomes (11, 12).

Outcome expectations refer to a person's beliefs about what results or consequences their actions will have. It includes the idea that certain actions will lead to certain outcomes. These expectations influence a person's motivation to perform or avoid certain actions. Bandura describes outcome expectations briefly as “the outcomes people expect their actions to produce” (11), (p. 144). Bandura assumes a three-factor pattern that distinguishes between physical, social, and self-evaluative outcome expectations (8, 13). Physical beliefs refer to physical or health-related changes as a consequence of a behavior. For example, regular jogging could result in a slimmer figure or an improved immune system. Bandura defines social outcome expectations as the reactions of other people or groups of people provoked by a specific behavior. Examples of this are recognition or praise, but also condemnation and rejection by third parties. Self-evaluative beliefs include anticipated personal feelings as a result of an action. For example, an increased level of physical activity could be associated with feelings of happiness, satisfaction, and pride. Outcome expectations were shown to act as a mediator in the SCT model previously (14, 15).

Referring to Bandura's 1977 publication, Luszczynska and Schwarzer (16) (p. 132) claim that “Sociostructural factors refer to the impediments (barriers) or opportunities that reside in living conditions, health systems, political, economic or environmental systems”, which matches Bandura's brief description of sociostructural factors as the perceived facilitators and social and structural impediments (8). The impact of sociostructural factors on physical activity was demonstrated previously (17–19).

Finally, goals are specific outcomes or states that a person wants to achieve. They serve as motivating factors that influence a person's behavior and guide their actions in a specific direction. “Goals, rooted in a value system, provide further self-incentives and guides to health behavior. Goals may be distal ones that serve an orienting function, or proximal ones that regulate effort and guide action in the here and now.” (20), (p. 628). Previous research shows that goal setting has a direct effect on physical activity behavior (21), as assumed by Bandura.

**Figure 1:**
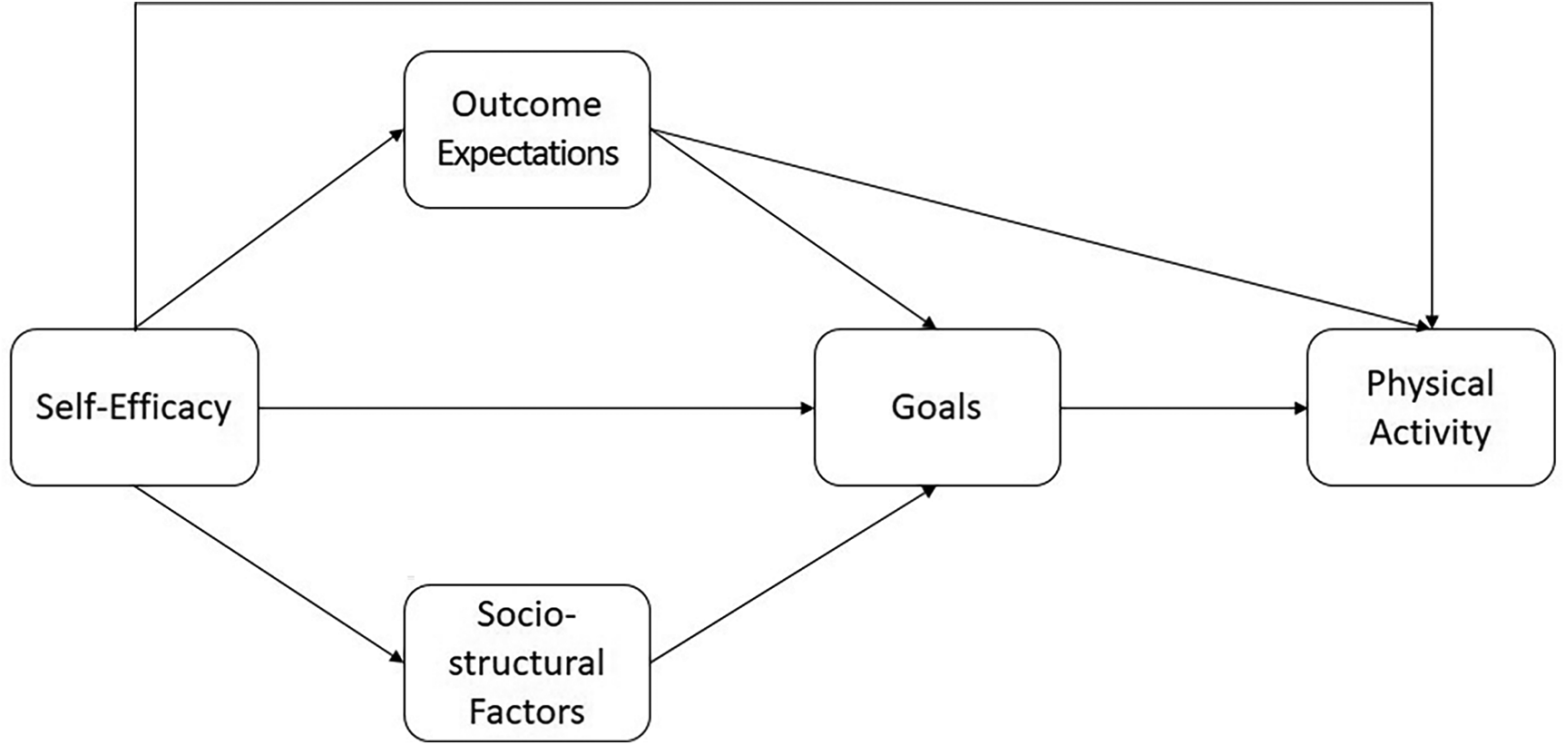
Social cognitive theory.

Figure 1 displays the interplay between the elements. According to Bandura (4), self-efficacy has a direct effect on outcome expectations, sociostructural factors, goals, and physical activity. Outcome expectations have a direct effect on physical activity and an indirect effect on physical activity via goals. Sociostructural factors have an indirect effect on physical activity via goals.

There is a broad range of literature that has tested and confirmed parts of SCT concerning physical activity, both in specific populations and in selected domains (22–25). Overall, research supports SCT as a sound basis for understanding and improving physical behavior (7, 11).

Yet, some limitations are evident, that may reduce the quality of the studies and thus the interpretability of the findings (11). In the context of this study, one limitation will be singled out: To assess elements of SCT, in the absence of suitable, matching, appropriate, and parsimonious validated scales to capture the elements, researchers usually create their own item sets to measure elements of the SCT (16). This is also a limitation frequently cited in meta-analyses on SCT. As observed by Young et al. (11), for example, a common issue in the research on SCT is the lack of adequate evidence to substantiate the reliability, let alone the validity, of the scales employed in the research process. They report: “only 4% of the models measured all SCT constructs using scales with adequate internal consistency and test–retest reliability” (p.15). Apart from the criticism that many studies thus far have only investigated individual elements of SCT, the main criticism here is that the findings are based on scales with unconfirmed psychometric properties.

The absence of coherent scales with established quality criteria is problematic in several respects. Firstly, with regard to the measurement of individual SCT constructs, the construct coverage of items in self-constructed scales is often uncertain, which makes the assessment of constructs more challenging and the quality criteria less reliable. This is exemplified by the work of Dewar et al. (26) and Peyman et al. (27), who constructed their own scales without subjecting them to the necessary validation procedures. On the one hand, it seems problematic that the construct coverage is only partially given. For example, Dewar and colleagues reduce socio-structural factors to parental support, and outcome expectations cover physical and social outcome expectations, but not self-evaluative outcome expectations. With Peyman and colleagues, the construct coverage is entirely unclear, as no further information is available about the items used. On the other hand, the quality of measurement is unclear, as the potential for measurement error in scales lacking essential quality criteria may result in the generation of inaccurate data. This can lead to the distortion of relationships between variables and a reduction in the statistical power of studies. Irrespective of whether self-created scales or already validated questionnaires are employed, it is imperative to accord due attention to criterion-related validity, that is to say, the congruence between the item formulation and the criterion in question. Such an issue is exemplified by the study conducted by Taymoori et al. (28), wherein the researchers recorded exercise-specific self-efficacy and outcome expectations, yet included physical activity in general as a criterion. A similar pattern is observed in the study by Smith et al. (29), where general physical activity-related self-efficacy and outcome expectations are recorded, but the number of steps was used as a criterion. This raises questions about the criterion-related validity of the items.

Secondly, the utilization of self-constructed scales or the assembly of multiple previously validated scales also represents a significant challenge when examining multiple elements of the SCT within a single study. In such instances, it becomes difficult to ascertain the extent to which the observed results, such as the relations of the constructs, can be attributed to the item sets or to the underlying constructs themselves. This limitation is exemplified by studies conducted by Gellert et al. (30), and Petosa et al. (31), as well as Plotnikoff et al. (32), which employ disparate questionnaires to assess various SCT constructs. While it is encouraging that the authors utilize previously validated scales, the correlations and overlaps of the constructs underlying the respective scales remain unclear.

As a result, the findings of previous studies are difficult to compare. While different studies may ostensibly capture the same construct, as long as the items are different and their quality criteria are unclear, the findings can only be interpreted with caution. Therefore, it is highly critical when researchers develop their own scales or combine different scales to measure constructs of SCT.

The two aspects of the recording problem are now evident: firstly, there is a need for meaningful scales that encompass the discrete elements; secondly, there is a requirement to ascertain the interrelationships between the individual scales to enable the recording of several constructs of SCT in one study.

In light of the dearth of empirical evidence pertaining to all four tenets of SCT, the availability of a comprehensive questionnaire that incorporates all four elements seems of paramount importance to rule out potential effects of poor psychometric properties of the assessment.

Several validated scales have been developed which appear to be well suited for recording individual SCT components. For example, the Exercise Self-Efficacy Scale (33) or the Multidimensional Outcome Expectation for Exercise Scale (15) could be used as a foundation for further development. However, we elected to eschew this approach for three reasons. Primarily, the scales are relatively dated (33), have not been validated in German-speaking countries (15), and do not align with the intended criterion – physical activity as defined by the WHO as physical activity in general, not a specific physical activity behavior (such as jogging, number of steps, etc.) (2).

Therefore, it is our goal to develop a parsimonious, reliable, and valid questionnaire to assess the four elements of SCT in the context of physical activity behavior. The objective of this study is not to develop a comprehensive scale that can replace all existing scales created by other authors. Instead, the aim is to develop a concise questionnaire that meets defined quality criteria and can be used in conjunction with more detailed scales if necessary. To enhance clarity and readability, we have divided the objective into two parts and will present the respective methods and results separately. Part 1 of this study aims at selecting items for the assessment of the elements of SCT from an initial item pool and testing their factorial structure. The aim of Part 2 is to examine the internal consistency, and test-retest reliability, as well as evidence for convergent and discriminant validity.

## 2 Part 1: item selection

### 2.1 Materials and methods

#### 2.1.1 Sample

Before data collection, we conducted a theory-based sample planning. According to Tay and Jebb (34), an initial sample of about 100 participants should be acquired to examine the psychometric properties of the items and about 300 participants should form the confirmatory sample. Therefore, the target sample size was 400 participants minimum for the first measurement point. The study was open to subjects aged between 18 and 64 years old, with a command of the German language at the native speaker level and no health restrictions that would preclude participation in physical activity. In total, 470 participants answered the first questionnaire, (191 male, 278 female, 1 diverse) participants with a mean age of 33 years (SD = 12.94, Range 18–65). Participants who dropped out of the questionnaire in the middle were excluded from the analyses for the scale. This affected a total of 36 test subjects. After excluding the dropouts, the sample consisted of 434 participants [173 male (39.9%), 260 female (59.9%), 1 diverse (.2%)] participants with a mean age of 32.42 years (SD = 12.71, Range 18–65). The majority of respondents reported that they had completed upper secondary school or had completed higher education (75%). To analyze data, the sample was split into two random halves. Data from the first subsample was used to reduce the item pool. Data from the second subsample served to confirm the findings of the first subsample. The first subsample consisted of about 25% of participants [*n* = 109, 41 male (37.6%), 68 female (62.4%)] with a mean age of 34.81 years (SD = 13.95), the second subsample consisted of 325 participants [132 male (40.6%), 192 female (59.1%)] with a mean age of 31.62 years (SD = 12.18). Comparability of the two subsamples was ensured concerning socio-demographic characteristics and physical activity (all *p* > .2), except for age, where the two subsamples differed significantly, *t* (432) = 2.28, *p* = .012, Cohen's *d* = .25.

#### 2.1.2 Measures

As the constructs in question were already thoroughly defined by Bandura himself and refined over time by multiple authors, a deductive approach to item generation was applied as recommended in the literature (34). In total, four item pools were developed – one for each element of the theory, following the definitions of the constructs given in the introduction. Existing scales were reviewed and additional items were generated by the authors based on the above-mentioned definitions where the authors did not see the above definitions covered. Established criteria for formulating items were followed (34). As the questionnaire is intended for use in the general adult population in Germany aged 18–64 years, too high situational specificity of the items was refrained from so that the items represent the physical activity behavior of the average population. Thus, the behavior to be explained and predicted is physical activity in general, not a specific physical activity behavior (such as jogging, number of steps, etc.). Thereby, we followed the distinction of the World Health Organisation of physical activity and exercise, where exercise is seen as part of physical activity, but physical activity is not limited to exercise but also includes walking, everyday movements, etc. (2).

The original item pool in German as well as an English translation can be found in Supplementary Material 1. As suggested by Tay and Jebb (10), issues of redundancy were neglected as they might serve to uncover facets of the constructs.

The instrument is intended for use in research on the theory and interventions based on the theory in the general adult population in Germany aged 18–64 years. The purpose of the questionnaire guided the item creation as it determined the appropriate reading level of the sample, the applicability of reverse scoring, the specificity of the contexts and situations given in the items, and the response format. Data from the OECD confirm that German adults have an average reading proficiency (35). Too high situational specificity of the items was refrained from so that the items represent the physical activity behavior of the average population. Concerning the response format, we refrained from using a “don't know” fallback category, since it is particularly suitable when it must be assumed that individual subjects do not have the skills to answer the question. In the case of SCT, it can be assumed that the subjects are very well able to estimate the answers to the questions. Regarding the polarity of the scale, a unipolar graded Likert scale was used for the questionnaire, on which agreement was expressed from “very little” (0) to “very much” (10). Thereby, we followed Bandura's suggestion for items concerning SCT. Bandura advocates for a sensitive scale and advises against scales with fewer choices (such as a five-point scale for example). Often, a 100-point unipolar rating scale is used to assess self-efficacy (36). As this response format is very demanding on respondents, we tried to strike a balance between high sensitivity and good responsiveness.

#### 2.1.3 Procedure

Data collection comprised two measurement time points set one week apart. The questionnaires were provided online using the SoSci software (37). The conduct of the study complied with the ethical standards of the responsible committee (anonymized for blind review). Written informed consent was obtained from all subjects before the study. Then, in the first questionnaire, participants answered questions about demographic data, personality, and physical activity. Then, participants answered the items created to assess self-efficacy, goal setting, outcome expectations, and sociostructural factors. Additional variables were assessed for discriminant and convergent validity. These will be described in more detail in Part 2. We randomized both the order of the constructs as well as the order of the items to minimize measurement error. In the second questionnaire, participants answered the same questionnaire except for demographic data and personality measures, which we only recorded once. For Part 1, only the data from the first measurement point was used.

#### 2.1.4 Statistical analyses

In the first step, items were reduced to a set of items with reasonable psychometric properties. We examined the mean, standard deviations, ranges, and item-total correlations for each item of the respective subscale. In total, 9 items were excluded from the item pool because their mean was too high (>8) or too low (<2) [2 self-efficacy (S), 3 outcome expectations (O), 4 sociostructural factors (F)], and 26 items were excluded from the item pool because their item-total correlation was too small (<.5) [16 O, 7 F, 3 goals (G)]. Details on these analyses can be found in the Supplementary Material 2. We also looked at the internal consistency of the scales. We aimed at an internal consistency of .70 minimum which was given for all four subscales.

To examine the dimensionality, a principal axis analysis with interrelated factors (oblimin rotation) was performed over all items for each subscale. Items were excluded if their loading on the assumed factors in the pattern matrix was below .40, if they built separate factors, or cross-loaded higher than .30 on other factors. Details on these individual exploratory factor analyses can be found in Supplementary Material 3. Then, to examine the dimensionality of the questionnaire, a principal axis analysis with interrelated factors (oblimin rotation) was performed over all remaining items. Again, items were deleted from the item pool following the above-mentioned criteria. Details on this analysis can be found in Supplementary Material 4. In addition to the statistical analysis, the remaining items were then reviewed by an independent expert concerning construct coverage and item formulation. In the event of concerns, another factor analysis would be conducted as described above after eliminating items.

In the second step, a confirmatory factor analysis was conducted in the second subsample based on the items that were selected in the first subsample. We examined the comparative fit index (CFI), Tucker-Lewis index (TLI), root mean square error of approximation (RMSEA), and standardized root mean square residual (SRMR). General standards (e.g., 20) hold that the minimum standards of a good fit for these metrics are: CFI ≥ .90, TLI ≥ .90, RMSEA ≤ .08, and SRMR ≤ .08 (34, 38).

Data were analyzed using IBM SPSS Statistics Version 29 and JASP Version 0.18.3.0. Missing values were treated via listwise deletion.

### 2.2 Results

For reasons of space, solely the final factor analysis and subsequent analyses are reported in the manuscript. All preparatory analyses, as described in the Statistical analyses section, are documented in the Supplementary Material.

A principal axis factor analysis was conducted on the 18 items with oblique rotation (direct oblimin). The Kaiser-Meyer-Oklin measure was .840, categorized as meritorious by Hutcheson and Sofroniou (39). An initial analysis was run to obtain eigenvalues for each factor of the data. 4 factors had eigenvalues over Kaiseŕs criterion of 1 and in combination explained 64% of the variance. Table 1 shows the factor loadings after rotation. The items that cluster on the same factor suggest that factor 1 represents self-efficacy, factor 2 represents sociostructural factors, factor 3 represents goals and factor 4 represents outcome expectations.

**Table 1:** Pattern matrix.

	Factor			
	1	2	3	4
S1 Ich bin dazu in der Lage, mich auch dann zu bewegen, wenn ich zuhause viel zu tun habe.	0.941			
S2 Ich bin dazu in der Lage, mir eine Routine aufzubauen, mich regelmäßig zu bewegen.	0.884			
S3 Ich bin dazu in der Lage, mich auch dann zu bewegen, wenn in meinem Leben viele Dinge passieren.	0.851			
S4 Ich bin dazu in der Lage, mich zu bewegen, obwohl ich andere zeitliche Verpflichtungen habe.	0.770			
S5 Ich bin dazu in der Lage, mich zu bewegen, obwohl das Wetter schlecht ist.	0.675			
F1 Mangelnde soziale Unterstützung hindert mich daran, mich zu bewegen.		0.932		
F2 Mangelnde Anreize hindern mich daran, mich zu bewegen.		0.733		
F3 Körperliche Einschränkungen hindern mich daran, mich zu bewegen.		0.675		
F4 Familiäre Verpflichtungen hindern mich daran, mich zu bewegen.		0.510		
G1 Ich setze mir bewegungsbezogene Ziele.			0.944	
G2 Ich setze mir kurzfristige bewegungsbezogene Ziele.			0.880	
G3 Ich setze mir langfristige bewegungsbezogene Ziele.			0.787	
G4 Ich habe ein konkretes bewegungsbezogenes Ziel.			0.589	
O1 Bewegung stärkt meine Knochen.				0.844
O2 Bewegung kann Verletzungen vorbeugen.				0.745
O3 Bewegung hilft mir dabei, mit Stress umzugehen.				0.497
O4 Bewegung hilft mir dabei, selbstbewusst zu sein.				0.496
O5 Bewegung hilft mir dabei, mein Gewicht zu halten.				0.460

S = self-efficacy, F = sociostructural factors, G = goals, O = outcome expectations.

Based on these items, a confirmatory factor analysis was conducted in the second subsample to confirm the factorial structure. The four-factor model resulted in a good fit with *χ*^2^ (129) = 330.06, *p* < .001; RMSEA = .069; CFI = .928; TLI = .915; SRMR = .074. The fit indices fulfill the requirements for a good model fit.

Table 2 displays the factor loadings. All items loaded significantly and highly on the assumed factors.

**Table 2:** Factor loadings.

Factor	Indicator	Estimate	Std. error	*z*-value	*p*	95% confidence interval	
						Lower	Upper
Self-efficacy
	S1	2.457	0.116	21.117	<.001	2.229	2.685
	S2	1.873	0.128	14.638	<.001	1.622	2.124
	S3	2.277	0.111	20.440	<.001	2.058	2.495
	S4	2.118	0.122	17.312	<.001	1.878	2.358
	S5	1.724	0.134	12.870	<.001	1.461	1.986
Outcome expectations
	O1	1.461	0.140	10.400	<.001	1.186	1.736
	O2	1.283	0.135	9.503	<.001	1.018	1.548
	O3	1.538	0.137	11.254	<.001	1.270	1.806
	O4	1.761	0.140	12.607	<.001	1.487	2.035
	O5	1.489	0.154	9.694	<.001	1.188	1.790
Sociostructural factors
	F1	2.555	0.153	16.707	<.001	2.255	2.854
	F2	2.088	0.153	13.620	<.001	1.788	2.389
	F3	1.782	0.156	11.461	<.001	1.477	2.087
	F4	0.507	0.176	2.884	0.004	0.162	0.852
Goals
	G1	2.625	0.126	20.861	<.001	2.378	2.872
	G2	2.090	0.141	14.869	<.001	1.814	2.365
	G3	2.330	0.137	17.013	<.001	2.061	2.598
	G4	2.468	0.137	18.000	<.001	2.199	2.736

## 3 Part 2: reliability and validity

To assess the psychometric quality of the items developed, the internal consistency of the scales and the retest reliability will be evaluated. In terms of the internal consistency of the scales, the desired outcome is for the values to align with the established conventions, specifically a Cronbach's alpha score of greater than 0.7. Concerning retest reliability, it is anticipated that values will fall within the range of .60–.70 when comparable scale developments are used as a benchmark (40, 41).

To ascertain the validity of the developed scale, content-related validity, criterion-related validity, and construct-related validity were examined (42). To ensure content-related validity, an independent expert reviewed the construct coverage and face validity of the subscales. As the scale is to be developed with specific reference to the context of physical activity behavior, the bivariate correlations with physical activity behavior will be evaluated as an indicator of criterion-related validity. It is anticipated that moderate correlations will be observed in this instance, with positive associations emerging for self-efficacy, outcome expectations, and goals, and negative correlations for sociostructural factors. In light of the findings of previous research, which have reported particularly strong correlations between self-efficacy and outcome expectations with physical activity behavior for example summarized in the meta-analysis of Young et al. (11), we hypothesize that the relationship between these constructs and physical activity behavior will be stronger than the relationship between physical activity and sociostructural factors. Concerning goals, a positive correlation is anticipated, although the strength of this association remains uncertain due to the presence of conflicting evidence. Some researchers have reported a strong relationship between goals and physical activity behavior (8), whereas others have highlighted the discrepancy between these two constructs and have assumed a random association between them (43).

Ensuring content-related validity entailed determining whether the subscales exhibited the anticipated correlations with analogous constructs or demonstrated low correlations with constructs with which a lower conceptual agreement is assumed. Before data collection, literature research was conducted to determine which constructs are conceptually close to the elements of SCT and which constructs differ from each other. Accordingly, the following correlations were assumed as the basis for the validity of the developed scale.

It is anticipated that the four subscales will demonstrate a moderate correlation with one another. It is assumed that self-efficacy, outcome expectations, and goals are positively correlated, whereas negative correlations are expected with sociostructural factors, given the conceptualization of sociostructural factors as barriers to exercise. Moderate correlations are interpreted as evidence of discriminant validity, indicating that the four subscales capture different aspects. In light of prior research findings, it is plausible that certain subscales may exhibit a stronger association with one another than with other subscales. For instance, it is conceivable that the correlations with socio-structural factors may be less pronounced than those observed with self-efficacy and outcome expectations (11).

Moreover, following Bandura's construct definitions and delimitations, the self-efficacy subscale is anticipated to be associated with general self-efficacy (9, 36). Bandura would suggest that a small to moderate correlation is to be expected, given that specific self-efficacy may differ from general self-efficacy. It is therefore anticipated that a small, positive correlation will be observed. Given the conceptual proximity of general and specific self-efficacy, it is expected that the remaining three SCT constructs will also demonstrate a small correlation with general self-efficacy, with positive associations for outcome expectations and goals and negative associations for sociostructural factors. However, it is acknowledged that the correlations may be smaller than the relations of the three constructs with the self-efficacy subscale, given that this facet of self-efficacy is specifically tailored to physical activity.

Further evidence for convergent and discriminant validity could be provided by low correlations of physical activity-specific self-efficacy with self-esteem and internal locus of control, as Bandura differentiates these three constructs from each other (17). Whereas self-efficacy is a judgment of capability, self-esteem is a judgment of self-worth, and locus of control is concerned with the contingency of outcomes – whether the outcomes are caused by one's actions or by forces beyond one's control (17). Therefore, both self-esteem and internal locus of control should correlate with the self-efficacy subscale, but ideally not show high correlations with physical activity-specific self-efficacy. Low correlations could be interpreted in terms of convergent validity, as the three constructs are associated with one another. However, as Bandura delineates the constructs, small correlations might also indicate discriminant validity, demonstrating that the three constructs differ from one another.

A positive correlation between outcome expectations or goals and internal locus of control, or conversely, a negative correlation between outcome expectations or goals and external locus of control, could be postulated based on the construct definition of locus of control as a generalized expectation of internal or external reinforcement (44). This implies that internal locus of control may be defined as a conviction that one can control events and experiences as a consequence of one's behavior. However, there is a paucity of research on these relationships, particularly on the relationships between these constructs and socio-structural factors.

### 3.1 Materials and methods

#### 3.1.1 Sample

Irrespective of the division of the sample in Part 1, all participants who had completed both measurement points were included in this sample. Due to dropout and missing data, data from 196 participants was used for repeated measures [47 male (24%), 148 female (75.5%), and 1 diverse (0.5%)] with a mean age of 26.17 years (SD = 10.51). Dropout analyses showed no significant differences between participants who did not take the second measurement point and those who did concerning their scores on the subscales and their education level (all *p*'s > .2). However, more men than women dropped out, *χ*^2^ (1) = 3.82, *p* = .051, and participants dropping out were significantly older (*M* = 39.24, SD = 11.11) than those remaining for the second measurement (*M* = 27.20, SD = 8.70), *t* (323) = 2.37, *p* = .01.

#### 3.1.2 Measures

Self-efficacy, outcome expectations, sociostructural factors, and goals were assessed with the final subscales created in Part 1. Physical activity was assessed by seven items of the International Physical Activity Questionnaire – Short Form in German [IPAQ-SF, (45)]. The IPAQ-SF assesses the number of days and the average time (hours and minutes) spent on physical activity with an open response format. IPAQ-SF was chosen because of its parsimony, its good psychometric qualities, and its implementation in multiple previous studies (46, 47). General self-efficacy was assessed using the General Self-Efficacy Scale (SWE) (48). This is a four-point, unidimensional scale consisting of ten items. To assess the locus of control, the “Skala Internale-Externale Kontrollüberzeugungen-4” (44) was used. This rating scale consists of four items that assess internal and external locus of control. Self-esteem was assessed by Robins et al. (49) Single-Item Self-Esteem Scale. Big Five Inventory [BFI-10, (50)] was used to assess personality dimensions according to the five-factor model by 10 items.

#### 3.1.3 Procedure

#### 3.1.4 Statistical analyses

To examine the reliability of the scales, Cronbach´s Alpha was examined for internal consistency of the subscales and repeated measures Pearson correlations were examined for retest-reliability. Convergent validity was tested by bivariate correlations of the subscales with physical activity. Moreover, the bivariate correlation of the self-efficacy subscale and general self-efficacy was examined for convergent validity. Discriminant validity was tested by bivariate correlations of the subscales with each other. Additionally, the bivariate correlations of the self-efficacy subscale with the locus of control and self-esteem were tested.

### 3.2 Results

Table 3 shows Cronbach's Alphas, retest-reliability, means, standard deviations, and ranges derived from the second subsample for each of the four subscales. Table 4 shows bivariate correlations derived from the second subsample for each of the four subscales and further constructs.

**Table 3:** Cronbach's Alphas, retest-reliability, means, standard deviations, and ranges.

Scale	Cronbach's Alpha	Retest-reliability	Mean	Standard deviation	Range
Self-efficacy	.899	.768**	5.25	2.21	0–9
Outcome expectations	.746	.770**	6.08	1.75	0.6–9
Sociostructural factors	.691	.735**	6.31	2.05	0–9
Goals	.890	.700**	5.19	2.51	0–9

*N* = 325 except for retest-reliability, where *N* = 196 due to dropout.

** = *p* < .001.

**Table 4:** Bivariate correlations.

Scale	Self-efficacy	Outcome expectations	Sociostructural factors	Goals
Self-efficacy	–			
Outcome expectations	.494**	–		
Sociostructural factors	.138*	.081	–	
Goals	.457**	.443**	-.076	–
Physical activity	.248**	.162**	.040	.185**
General self-efficacy	.274**	.314**	.151**	.182**
Self-esteem	.213**	.225**	.033	.089
Internal locus of control	.224**	.344**	.103	.089
External locus of control	−.196**	−.251**	−.308**	.065

*N* = 325, * = *p* < .05, ** = *p* < .001.

## 4 Discussion

Findings on SCT in the context of physical activity seem promising. However, they are limited concerning their interpretability (11) since researchers usually employ their own scales or sets of items to measure elements of the SCT (16). Therefore the goal of this study was to develop a parsimonious, reliable, and valid questionnaire to assess the elements of SCT in the context of physical activity. In Part 1, items were developed and tested for the four subscales of the theory. In Part 2, reliability and validity were examined.

The expected four-factor structure of the created items was confirmed in the confirmatory sample with a good model fit. The internal consistency of the four subscales was satisfactory, too. One exception is the socio-structural factors scale - here the internal consistency was just below the level at which internal consistency would be described as acceptable. However, this may be due to the construct definition, which appears to be more heterogeneous for socio-structural factors than for the other three constructs. That is, the items are not highly consistent simply because the construct to be measured is relatively heterogeneous. However, given the goal to generate parsimonious scales, internal consistency of the subscales can be considered satisfactory, since the number of items (4 or 5 items in the case of our subscales) is known to influence Cronbach's alpha. Here, a clear trade-off is given: By including more (reliable) items, the reliability of this scale easily could be increased, but only at the cost of economy, and this was an important goal in the context of our scale construction. This is widely recognized in health research and retest-reliability is considered to be of greater consequence than internal consistency (51).

Retest reliability was satisfactory (all *r* ≥ .70) for the four subscales. This is consistent with the retest reliability of comparable scales [e.g., (40, 41)]. It should be noted, however, that the shorter time span chosen for the retest interval may have affected the results. When determining the retest interval, it is necessary to strike a balance between a shorter interval, where there is a risk that subjects will remember their answers and the actual stability of the measurement cannot be clearly determined, and the possibility of an actual change in the trait to be measured (42, 51). Given the specificity of the cognitive variables, the underlying assumption of a state seems more likely than the assumption of a trait, which in turn would be more accurate for omnibus measures like general self-efficacy (9). However, considering the relatively short interval between the two measurements, participants may have remembered their answers to the first assessment and have felt a tendency to respond equally in the second assessment. If that was the case, retest reliability would have been overestimated and real retest reliability might be somewhat smaller. However, some authors claim retest reliability of .60 still as satisfactory (e.g., 24, 34), and it would therefore not be a cause for concern if the retest reliability was somewhat lower than reported. It would, however, be beneficial to conduct the retest reliability test again, in accordance with the recommended retest interval of 2–4 weeks, in order to gain a more accurate understanding of the values.

The four subscales were shown to be distinct by looking at their correlations (all |*r*| < .5). It is noticeable that self-efficacy and outcome expectations (*r* = .494) resp. goals (*r* = .457) correlate relatively highly with each other, which fits Bandura's theoretical assumptions. At the same time, however, it also shows once again that these are distinct constructs. Further bivariate correlations mostly confirmed the expected correlation pattern. These findings align with the content-related validity examined by an independent expert who evaluated the items in terms of their alignment with the specified constructs and determined that the final items demonstrated satisfactory construct coverage. Concerning criterion-related validity, as expected, the newly developed subscales correlated substantially with physical activity, except for socio-structural factors. It is noteworthy that a correlation was not found between socio-structural factors and physical activity. On the surface, the lack of a direct correlation between socio-structural factors and physical activity appears to align with Bandura's assertion of no direct effect of socio-structural factors on physical activity. However, the lack of significance in the correlation also suggests the absence of a relationship between the two variables via a third variable, which would have been assumed by Bandura in the indirect effect of socio-structural factors on physical activity through goals. It is conceivable that the impact of socio-structural factors in Germany is less pronounced than the other three components of the theory on physical activity behavior. However, there is currently no evidence to support this assumption, as studies on SCT in the physical activity context in Germany (35) did not include the socio-structural factors in the SCT model and cross-cultural research on SCT in the context of physical activity including Germany are scarce. It would therefore be desirable to compare our findings with those of future studies that carry out a holistic model test in a German sample. The dearth of evidence regarding a significant direct or indirect effect of sociostructural factors on physical activity behavior is also a matter of concern in the international context, as evidenced by meta-analyses (16), seemingly contradicting the hypothesis that in pluralistic cultures, sociostructural factors might be of greater importance than in individualistic cultures (52). It is, however, possible that the lack of relation between sociostructural factors and physical activity is a sampling artifact of the scientific studies themselves. For example, it is likely that people with extremely unfavorable socio-structural conditions do not take part in such studies, which makes it difficult to prove a corresponding effect due to the limited variance. It would be prudent for future studies to concentrate on the cultural aspect, while also ensuring that broader socio-economic milieus are taken into account. This would help to avoid prematurely dismissing the theoretical assumptions of the theory in question.

Concerning construct-related validity, all newly developed subscales correlated substantially with general self-efficacy. Small to moderately significant correlations were found here, which can be well substantiated theoretically, as a correlation between general self-efficacy and specific self-efficacy, outcome expectations, goals, and socio-structural factors is plausible. As expected, the correlations of the SCT constructs with general self-efficacy were smaller than those with the self-efficacy subscale specifically tailored to physical activity. The correlation between general and specific self-efficacy might have been higher in terms of convergent validity. However, Bandura (9, 36) suggests that a conceptual difference between general and specific self-efficacy is recognized, which in turn would explain why the correlation is not strong.

As hypothesized, the significant but modest correlations between the self-efficacy subscale and internal locus of control, resp. self-confidence support the theoretical notion of proximity yet differential proximity. Concerning external locus of control, negative relations were expected concerning self-efficacy, outcome expectations, and goals. Negative relations were found for self-efficacy and outcome expectations, as well as sociostructural factors, however, no significant relation was found for external locus of control and goals.

### 4.1 Limitations

As a limitation, it must be noted that, even though best endeavors were made to obtain a representative sample, the measurement quality and fit of the items for younger and older participants (<18, >65 years) would have to be confirmed in further studies. In addition, evidence on convergent and discriminant validity should be examined in more detail in future studies to both confirm and extend our findings. The recently devised scale should be subjected to a more comprehensive comparison with other existing inventories. In the initial stage of scale creation, our objective was to provide a broad delineation of the constructs. We recommended that further testing be conducted in the future. For example, it would be beneficial to ascertain whether the self-efficacy subscale correlates with the sources of self-efficacy. Concerning outcome expectations, it would also be possible to ascertain whether the subscale is related to specific scales for recording outcome expectations. This naturally presents a challenge when attempting to compare scales that have not been evaluated in terms of their psychometric quality. Nevertheless, such comparisons could prove beneficial in terms of validating the newly created scale and the scales used for comparison.

Further, it would be beneficial to investigate closer any correlations that have (or have not) been demonstrated in the context of convergent and discriminant validity. For instance, it would be beneficial to ascertain whether socio-structural factors are genuinely unrelated to physical activity behavior, or whether our subscale was merely not successful to demonstrate this. Furthermore, the link between goals and behavior should be investigated in greater depth, particularly given the well-documented intention-behavior gap.

A final limitation concerns our scale construction. As we aimed to construct a parsimonious scale, construct coverage is now only given in the central aspects of the constructs, an observation that was also mentioned by the independent expert in the context of content validity. Specific facets are not covered by our scales. For example, outcome expectations can be divided into three subcategories: physical, affective, and social (Bandura, 1996). However, the items pertaining to social outcome expectations were excluded from the item pool during scale development due to the limited selectivity observed. Furthermore, the scale may lack sufficient differentiation concerning the sociostructural factors, which are conceptualized in a highly heterogeneous manner in the literature. Therefore, the possibility must be acknowledged that the developed scale does not fully reflect the complexity of the theory. Accordingly, despite this not being the aim of this study, it should be noted for future studies that - if the studies deal with specific aspects of the individual constructs (e.g., different types of outcome expectations) – the present items are probably not sensitive enough and should be supplemented with additional specific items. The principal benefit of the recently devised scale is its parsimony, which renders it suitable for utilization in prospective investigations of SCT in the context of physical activity. This may entail its deployment as an adjunct to more detailed scales, or as a means of ensuring comparability. Furthermore, with respect to the applicability of the scale in other language areas and countries, it should be noted that the scale was developed for the German language area and that the item selection and corresponding construct coverage may have been selective in this regard. It is conceivable that other socio-structural factors may be relevant in other countries (e.g., the healthcare system, and healthcare policy), which are not included in our scale because they do not play a differential role in Germany.

### 4.2 Conclusion

The parsimonious and valid instrument created to assess SCT in the context of physical activity hopefully enriches future research on SCT by providing short scales of which quality criteria are known and improving the quality of future studies on SCT in the context of physical activity and fostering comparability of the results across different studies in the future. Nevertheless, it must be demonstrated that the instrument functions effectively in a practical context, as the recently developed scale might also be used in clinical practice or health care as a screening instrument for professionals to ascertain pertinent information for treatment planning. Furthermore, the scale's brevity and straightforward language make it suitable for use by private individuals as a self-reflection tool, enabling them to assess their own physical activity behavior and its motivational aspects.

## Data Availability

The raw data supporting the conclusions of this article will be made available by the authors, without undue reservation.
