# Supplementary material for: Operationalization of the social cognitive theory to explain and predict physical activity in Germany: a scale development
Source: Front Sports Act Living. 2024 Nov 26;6:1508602. doi: 10.3389/fspor.2024.1508602 (PMC11628279; doi:10.3389/fspor.2024.1508602)
Supplement: Supplementary file 1 [file Datasheet1.docx]

Supplementary Material 1: Items

Initial item pool

1. **Self-efficacy**
   1. Ich bin dazu in der Lage, für kurze Strecken das Fahrrad zu nehmen.
   2. Ich bin dazu in der Lage, häufiger Strecken zu Fuß zu gehen.
   3. Ich bin dazu in der Lage, meinen Alltag so zu strukturieren, dass Bewegung einen Platz hat.
   4. Ich bin dazu in der Lage, mir eine Routine aufzubauen, mich regelmäßig zu bewegen.
   5. Ich bin dazu in der Lage, mich zu bewegen, obwohl das Wetter schlecht ist.
   6. Ich bin dazu in der Lage, mich auch dann zu bewegen, wenn in meinem Leben viele Dinge passieren.
   7. Ich bin dazu in der Lage, mich auch dann zu bewegen, wenn ich zuhause viel zu tun habe.
   8. Ich bin dazu in der Lage, mich nach der Arbeit zu bewegen.
   9. Ich bin dazu in der Lage, mich zu bewegen, obwohl ich andere zeitliche Verpflichtungen habe.
   10. Ich bin dazu in der Lage, mich zu bewegen, wenn ich emotionale Probleme habe.
   11. Ich bin dazu in der Lage, mich zu bewegen, wenn ich müde bin.
   12. Ich bin dazu in der Lage, mich zu bewegen, wenn ich mich durch die Arbeit unter Druck gesetzt fühle.
   13. Ich bin dazu in der Lage, mich trotz einer stressigen Lebenssituation ausreichend zu bewegen.
   14. Ich bin dazu in der Lage, mich trotz familiärer Probleme zu bewegen.
   15. Ich bin dazu in der Lage, mich trotz körperlicher Probleme zu bewegen.
   16. Ich bin dazu in der Lage, mich trotz langer Arbeitstage in meiner Freizeit zu bewegen.
   17. Ich bin dazu in der Lage, mich trotz meines Jobs ausreichend zu bewegen.
   18. Ich bin dazu in der Lage, mich trotz persönlicher Probleme zu bewegen.
   19. Ich bin dazu in der Lage, mich trotz widriger Umstände zu bewegen.
   20. Ich bin dazu in der Lage, mich vor der Arbeit zu bewegen.
   21. Ich bin dazu in der Lage, mich zu bewegen, auch wenn ich zu Hause viel zu tun habe.
2. **Outcome Expectations**
3. Bewegung gibt mir Energie.
4. Bewegung hilft mir dabei, eine positive Lebenseinstellung zu haben.
5. Bewegung hilft mir dabei, mein Gewicht zu halten.
6. Bewegung hilft mir dabei, meine täglichen Aufgaben zu bewältigen.
7. Bewegung hilft mir dabei, mich gut zu fühlen.
8. Bewegung hilft mir dabei, mich in meinem Körper wohlzufühlen.
9. Bewegung hilft mir dabei, mit Stress umzugehen.
10. Bewegung hilft mir dabei, Muskeln aufzubauen.
11. Bewegung hilft mir dabei, selbstbewusst zu sein.
12. Bewegung hilft mir dabei, wach und aufmerksam zu sein.
13. Bewegung hindert mich daran, mich mit meinen Freunden zu treffen. (R)
14. Bewegung hindert mich daran, Zeit mit meinen Freunden zu verbringen. (R)
15. Bewegung hindert mich daran, Zeit mit meiner Familie zu verbringen. (R)
16. Bewegung ist anstrengend für mich. (R)
17. Bewegung ist eine Möglichkeit, Zeit mit meinen Freunden zu verbringen.
18. Bewegung kann Verletzungen vorzubeugen.
19. Bewegung kostet mich viel Zeit. (R)
20. Bewegung nimmt zu viel meiner Zeit in Anspruch. (R)
21. Bewegung stärkt meine Knochen.
22. Bewegung unterstützt mein Herz-Kreislauf-System.
23. Bewegung verbessert meine Laune.
24. Bewegung verbessert meinen körperlichen Zustand.
25. Bewegung verbessert meinen mentalen Zustand.
26. Bewegung gibt mir das Gefühl, etwas geschafft zu haben.
27. Ich bin angenehm müde, nachdem ich mich bewegt habe.
28. Ich bin stolz auf mich, wenn ich mich bewegt habe.
29. Ich bin unangenehm müde, nachdem ich mich bewegt habe. (R)
30. Ich fühle mich gut, nachdem ich mich bewegt habe.
31. Ich fühle mich gut, wenn ich mich für meine Begriffe „ausreichend“ bewege.
32. Ich fühle mich schlecht, wenn ich meinen Bewegungsansprüchen nicht genüge. (R)
33. Ich fühle mich schlecht, wenn ich mich für meine Begriffe „zu wenig“bewege. (R)
34. Ich respektiere Menschen, die sich regelmäßig bewegen.
35. Meine Familie hält viel von Personen, die sich regelmäßig bewegen.
36. Meine Familie ist stolz auf mich, wenn ich mich ausreichend bewege.
37. Meine Freunde halten viel von Personen, die sich regelmäßig bewegen.
38. Meine Freunde sind stolz auf mich, wenn ich mich ausreichend bewege.
39. Muskelkater nach Bewegung reduziert mein Wohlbefinden. (R)
40. Meine Familie äußert sich positiv über meine körperliche Erscheinung.
41. Meine Familie belohnt mich dafür, dass ich mich bewege.
42. Meine Familie macht sich darüber lustig, dass ich mich bewege. (R)
43. Meine Freunde (Andere) äußern sich positiv über meine körperliche Erscheinung.
44. **Sociostructural Factors**
45. Das Wetter hält mich davon ab, mich zu bewegen.
46. Die Luftqualität hält mich davon ab, mich zu bewegen.
47. Familiäre Verpflichtungen hindern mich daran, mich zu bewegen.
48. Fehlende Bürgersteige halten mich davon ab, mich zu bewegen.
49. Körperliche Einschränkungen hindern mich daran, mich zu bewegen.
50. Lärm in meiner Umwelt hält mich davon ab, mich zu bewegen.
51. Mangelnde Angebote wie Fitnessstudios und Schwimmbäder halten mich davon ab, mich zu bewegen.
52. Mangelnde Sicherheit auf den Straßen hält mich davon ab, mich zu bewegen.
53. Mangelnde Sicherheit in meiner Umgebung hält mich davon ab, mich zu bewegen.
54. Mein Alter hindert mich daran, mich zu bewegen.
55. Mein Gewicht hindert mich daran, mich zu bewegen.
56. Mein mentaler Zustand hindert mich daran, mich zu bewegen.
57. Meine Arbeit hindert mich daran, mich zu bewegen.
58. Mangelnde soziale Unterstützung hindert mich daran, mich zu bewegen.
59. Mangelnde Anreize hindern mich daran, mich zu bewegen.
60. Meine Familie unterstützt mich dabei, dass ich mich genug bewege. (R)
61. Meine Freunde(Andere) bieten mir an, dass wir uns gemeinsam bewegen. (R)
62. Meine Freunde unterstützen mich dabei, dass ich mich genug bewege. (R)
63. **Goals**
64. Es ist mein Ziel, mich zu bewegen.
65. Ich habe das Ziel, mich täglich zu bewegen.
66. Ich habe ein konkretes bewegungsbezogenes Ziel.
67. Ich habe vor, mich zu bewegen.
68. Ich werde mich heute noch bewegen.
69. Ich werde mich im Laufe der Woche bewegen.
70. Ich werde mich im Laufe des nächsten Jahres mehr bewegen.
71. Ich setze mir bewegungsbezogene Ziele.
72. Ich setze mir kurzfristige bewegungsbezogene Ziele.
73. Ich setze mir langfristige bewegungsbezogene Ziele.

Final Item Pool

| Subscale | Items |
| --- | --- |
| Self-efficacy | 1. Ich bin dazu in der Lage, mich auch dann zu bewegen, wenn ich zuhause viel zu tun habe. 2. Ich bin dazu in der Lage, mir eine Routine aufzubauen, mich regelmäßig zu bewegen. 3. Ich bin dazu in der Lage, mich auch dann zu bewegen, wenn in meinem Leben viele Dinge passieren. 4. Ich bin dazu in der Lage, mich zu bewegen, obwohl ich andere zeitliche Verpflichtungen habe. 5. Ich bin dazu in der Lage, mich zu bewegen, obwohl das Wetter schlecht ist. |
| Sociostructural Factors | 1. Mangelnde soziale Unterstützung hindert mich daran, mich zu bewegen. 2. Körperliche Einschränkungen hindern mich daran, mich zu bewegen. 3. Mangelnde Anreize hindern mich daran, mich zu bewegen. 4. Familiäre Verpflichtungen hindern mich daran, mich zu bewegen. |
| Outcome Expectations | 1. Bewegung stärkt meine Knochen. 2. Bewegung kann Verletzungen vorbeugen. 3. Bewegung hilft mir dabei, mein Gewicht zu halten. 4. Bewegung hilft mir dabei, mit Stress umzugehen. 5. Bewegung hilft mir dabei, selbstbewusst zu sein. |
| Goals | 1. Ich setze mir bewegungsbezogene Ziele. 2. Ich setze mir kurzfristige bewegungsbezogene Ziele. 3. Ich setze mir langfristige bewegungsbezogene Ziele. 4. Ich habe ein konkretes bewegungsbezogenes Ziel. |

Translation

Initial item pool

1. **Self-efficacy**

1) I am able to ride my bike for short distances.

2) I am able to walk more frequently.

3) I am able to structure my everyday life in such a way that exercise has a place.

4) I am able to establish a routine of regular exercise.

5) I am able to exercise even though the weather is bad.

6) I am able to exercise even when there are many things going on in my life.

7) I am able to exercise even when I have a lot to do at home.

8) I am able to exercise after work.

9) I am able to exercise even though I have other time commitments.

10) I am able to exercise when I have emotional problems.

11) I am able to exercise when I am tired.

12) I am able to exercise when I feel under pressure at work.

13) I am able to get enough exercise despite a stressful life situation.

14) I am able to exercise despite family problems.

15) I am able to exercise despite physical problems.

16) I am able to exercise in my free time despite long working days.

17) I am able to get enough exercise despite my job.

18) I am able to exercise enough despite personal problems.

19) I am able to exercise despite adverse circumstances.

20) I am able to exercise before work.

21) I am able to exercise even if I have a lot to do at home.

1. **Outcome Expectations**

1) Exercise gives me energy.

2) Exercise helps me to have a positive outlook on life.

3) Exercise helps me to maintain my weight.

4) Exercise helps me to cope with my daily tasks.

5) Exercise helps me to feel good.

6) Exercise helps me to feel good in my body.

7) Exercise helps me to deal with stress.

8) Exercise helps me to build muscle.

9) Exercise helps me to be self-confident.

10) Exercise helps me to be awake and alert.

11) Exercise prevents me from socializing with my friends. (R)

12) Exercise prevents me from spending time with my friends. (R)

13) Exercise prevents me from spending time with my family. (R)

14) Exercise is exhausting for me. (R)

15) Exercise is a way to spend time with my friends.

16) Exercise can prevent injuries.

17) Exercise costs me a lot of time. (R)

18) Exercise takes up too much of my time. (R)

19) Exercise strengthens my bones.

20) Exercise supports my cardiovascular system.

21) Exercise improves my mood.

22) Exercise improves my physical condition.

23) Exercise improves my mental state.

24) Exercise gives me the feeling of having accomplished something.

25) I am pleasantly tired after exercise.

26) I am proud of myself when I have exercised.

27) I am uncomfortably tired after I have moved. (R)

28) I feel good after I have exercised.

29) I feel good when I move "sufficiently" for my needs.

30) I feel bad when I do not meet my exercise requirements. (R)

31) I feel bad when I move "too little" by my standards. (R)

32) I respect people who exercise regularly.

33) My family thinks highly of people who exercise regularly.

34) My family is proud of me when I get enough exercise.

35) My friends think a lot of people who exercise regularly.

36) My friends are proud of me when I get enough exercise.

37) Sore muscles after exercise reduce my well-being. (R)

38) My family makes positive comments about my physical appearance.

39) My family rewards me for exercising.

40) My family makes fun of the fact that I exercise. (R)

41) My friends (others) make positive comments about my physical appearance.

1. **Sociostructural Factors**

1) The weather prevents me from exercising.

2) The air quality prevents me from exercising.

3) Family commitments prevent me from exercising.

4) Lack of sidewalks prevents me from exercising.

5) Physical limitations prevent me from exercising.

6) Noise in my environment prevents me from exercising.

7) Lack of facilities such as gyms and swimming pools prevent me from exercising.

8) Lack of safety on the streets prevents me from exercising.

9) Lack of safety in my neighborhood prevents me from exercising.

10) My age prevents me from exercising.

11) My weight prevents me from exercising.

12) My mental state prevents me from exercising.

13) My work prevents me from exercising.

14) Lack of social support prevents me from exercising.

15) Lack of incentives prevents me from exercising.

16) My family supports me in getting enough exercise. (R)

17) My friends (others) offer me the opportunity to exercise together. (R)

18) My friends support me in getting enough exercise. (R)

1. **Goals**

1) It is my goal to exercise.

2) I have the goal of exercising every day.

3) I have a specific exercise-related goal.

4) I intend to exercise.

5) I will exercise today.

6) I will exercise during the week.

7) I will exercise more over the next year.

8) I set myself exercise-related goals.

9) I set myself short-term exercise-related goals.

10) I set myself long-term exercise-related goals.

Final Item Pool

| Subscale | Items |
| --- | --- |
| Self-efficacy | 1. I am able to exercise even when I have a lot to do at home. 2. I am able to establish a routine of exercising regularly. 3. I am able to exercise even when there are many things going on in my life. 4. I am able to exercise even though I have other time commitments. 5. I am able to exercise even though the weather is bad. |
| Sociostructural Factors | 1. Lack of social support prevents me from exercising. 2. Physical limitations prevent me from exercising. 3. Lack of incentives prevents me from exercising. 4. Family obligations prevent me from exercising. |
| Outcome Expectations | 1. Exercise strengthens my bones. 2. Exercise can prevent injuries. 3. Exercise helps me to maintain my weight. 4. Exercise helps me to deal with stress. 5. Exercise helps me to be self-confident. |
| Goals | 1. I set myself exercise-related goals. 2. I set myself short-term exercise-related goals. 3. I set myself long-term exercise-related goals. 4. I have a specific exercise-related goal. |
