# Supplementary material for: Operationalization of the social cognitive theory to explain and predict physical activity in Germany: a scale development
Source: Front Sports Act Living. 2024 Nov 26;6:1508602. doi: 10.3389/fspor.2024.1508602 (PMC11628279; doi:10.3389/fspor.2024.1508602)

Supplementary Material 2: ****Preliminary Item Analyses per construct****

**Table 1. Mean, standard deviation, and range for each self-efficacy item**
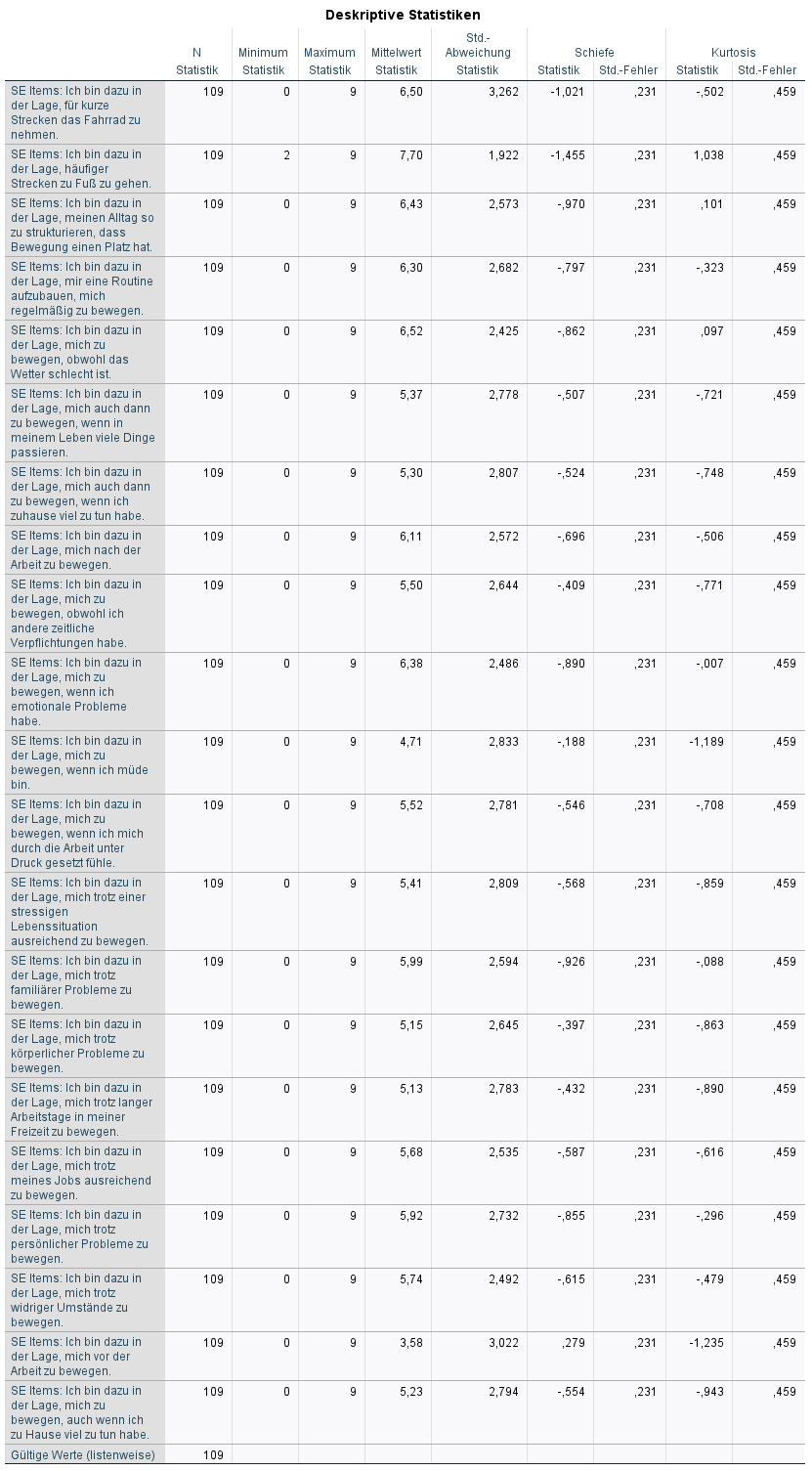


**Table 2. Item-total correlations for the self-efficacy items**
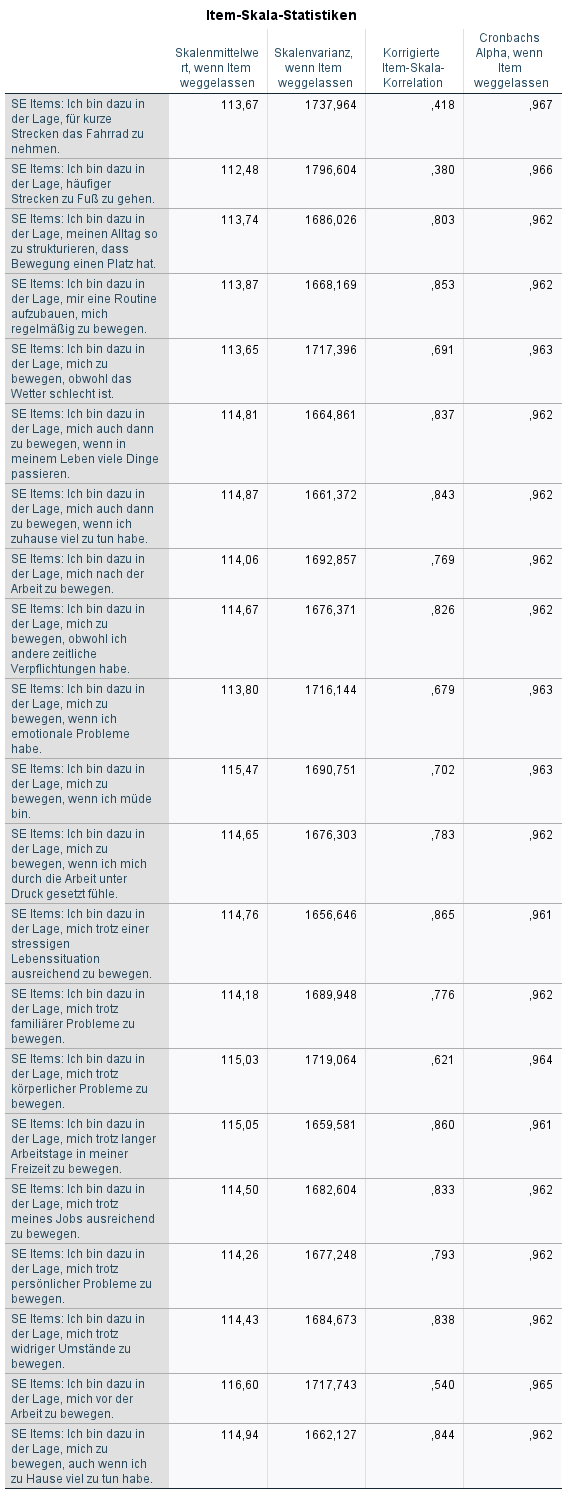


**Table 3. Mean, standard deviation, and range for each outcome expectations item**
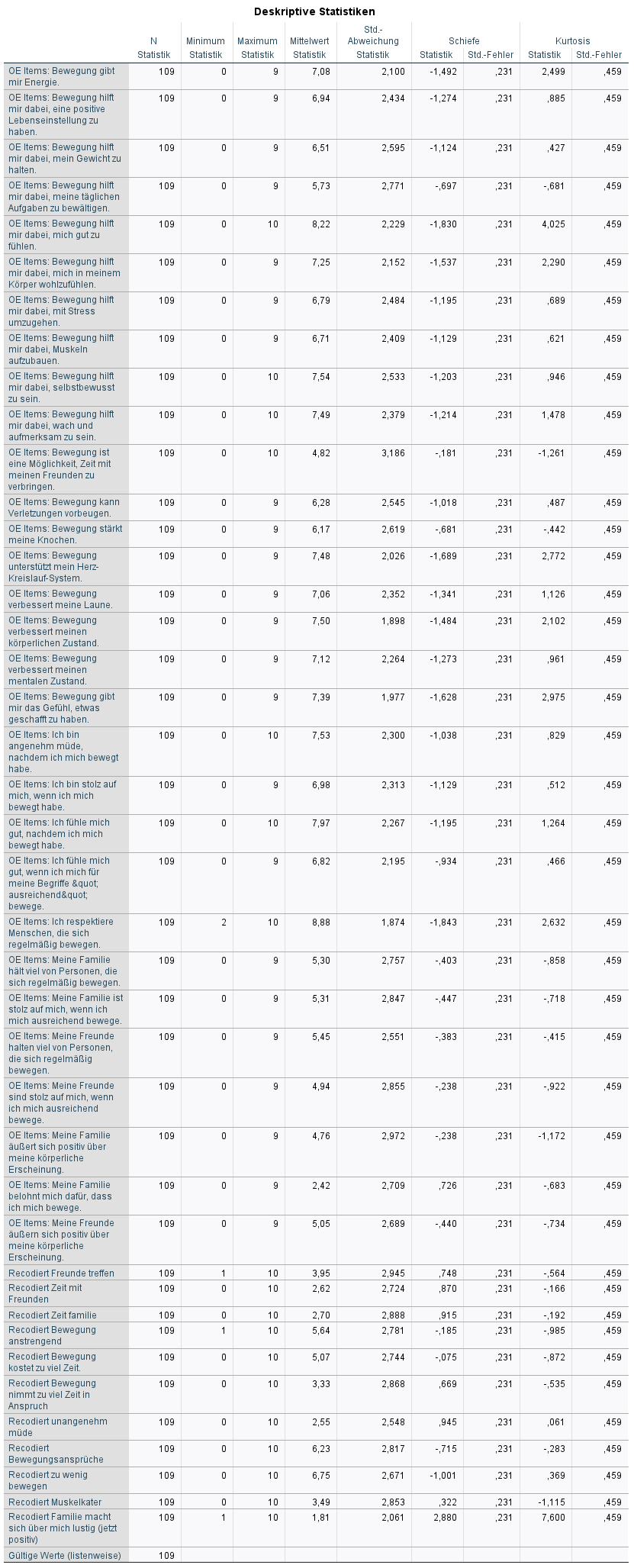


**Table 4. Item-total correlations for the outcome expectations items**
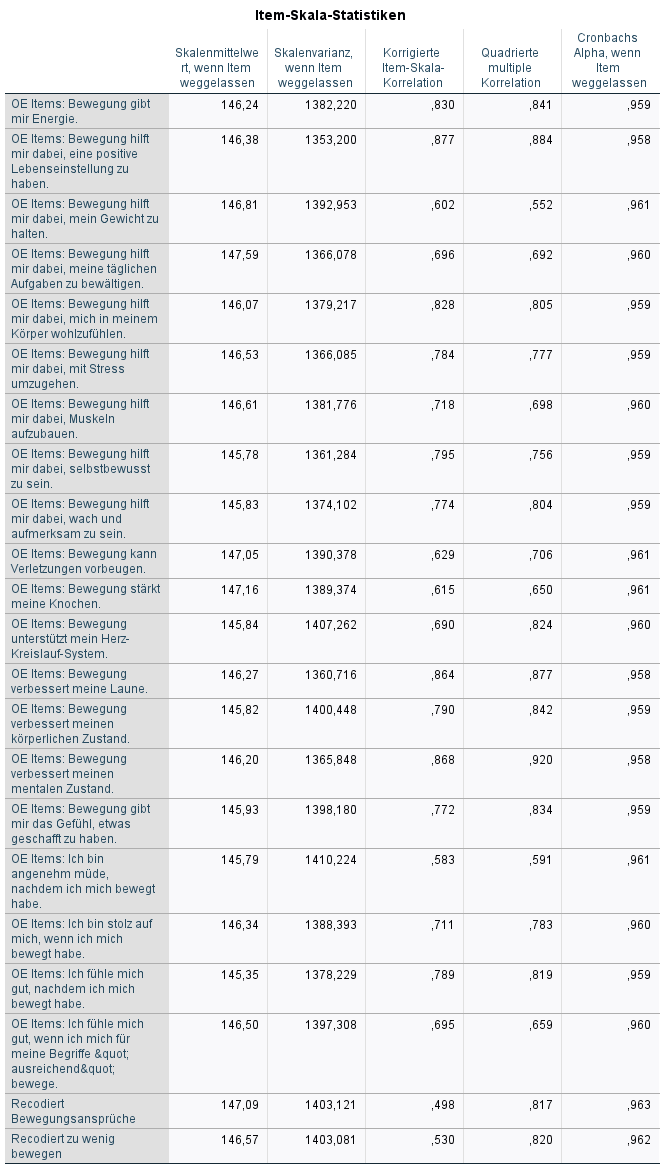


**Table 5. Mean, standard deviation, and range for each socio-structural factor item**


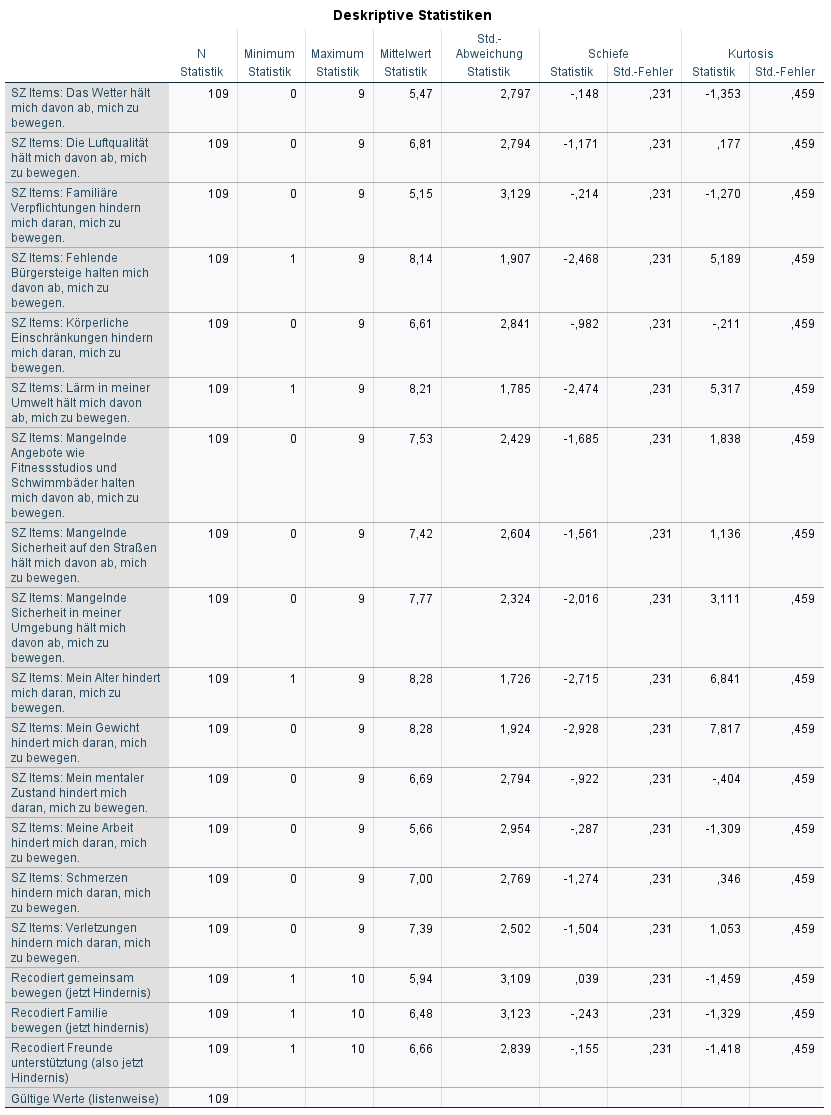


**Table 6. Item-total correlations for the socio-structural factor items**


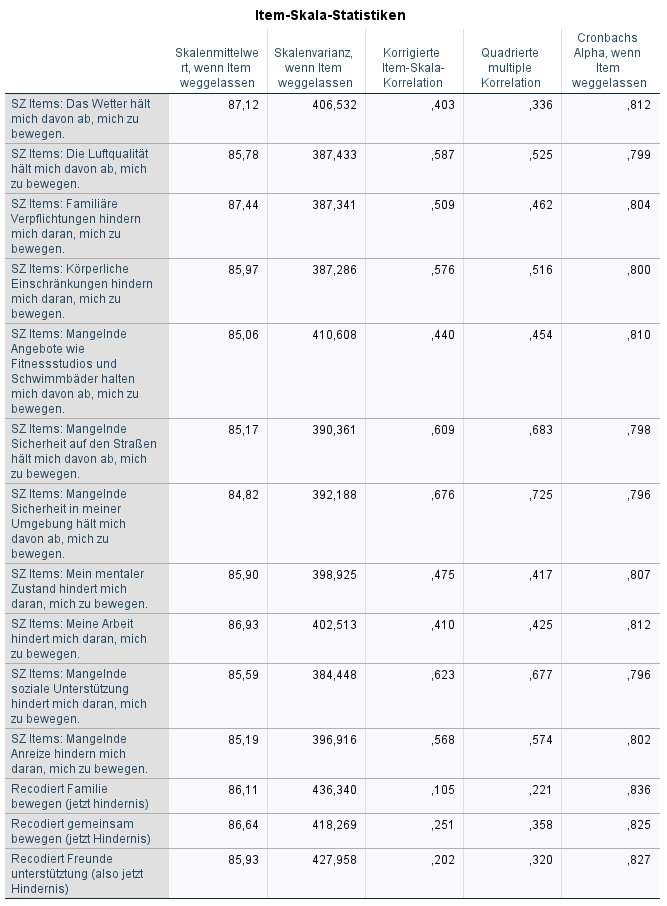


**Table 7. Mean, standard deviation, and range for each goal item**


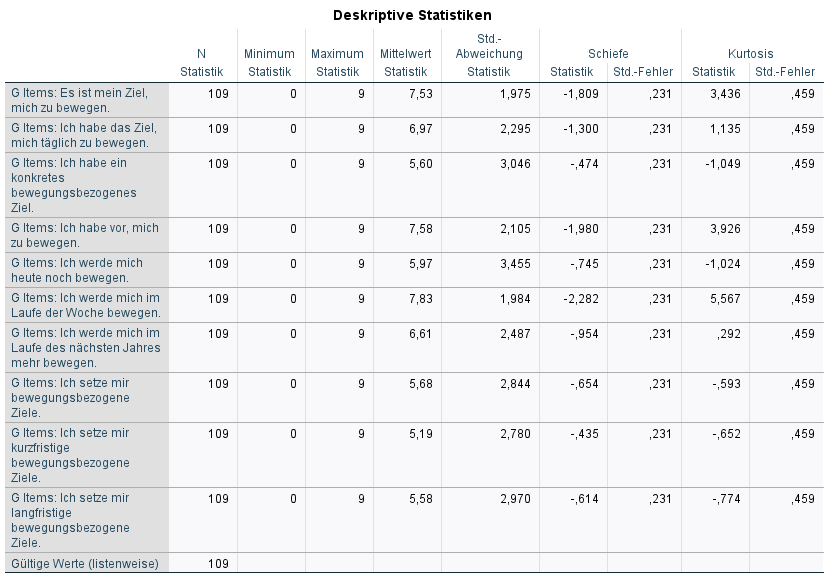


**Table 8. Item-total correlations for the goal items**


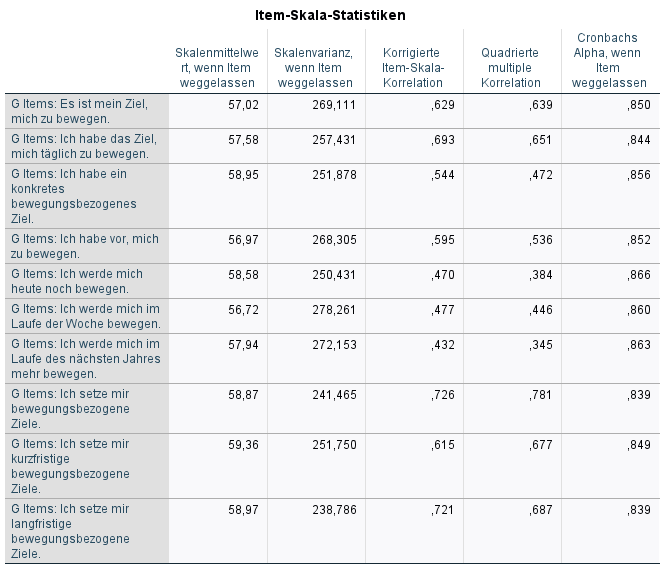

Supplement: Supplementary file 2 [file Datasheet2.docx]
