# Supplementary material for: Operationalization of the social cognitive theory to explain and predict physical activity in Germany: a scale development
Source: Front Sports Act Living. 2024 Nov 26;6:1508602. doi: 10.3389/fspor.2024.1508602 (PMC11628279; doi:10.3389/fspor.2024.1508602)

Supplementary Material 3: ****Preliminary Factor Analyses per construct****


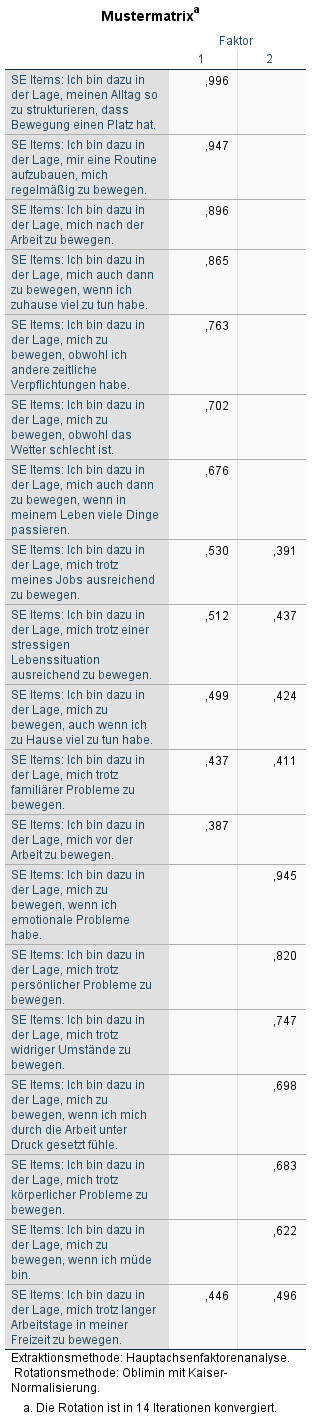


**Self-efficacy**

A principal axis factor analysis was conducted on the 19 items with oblique rotation (direct oblimin). The Kaiser-Meyer-Oklin measure was .918, categorized as marvellous by Hutcheson and Sofroniou (1999). An initial analysis was run to obtain eigenvalues for each factor of the data. 2 factors had eigenvalues over Kaiser´s criterion of 1 and in combination explained 69% of the variance.

Table 1 shows the factor loadings after rotation. The items that cluster on the same factor suggest that factor 1 represents self-efficacy related to external barriers to physical activity, factor 2 represents self-efficacy related to internal barriers to physical activity.

**Outcome Expectations**

A principal axis factor analysis was conducted on the 22 items with oblique rotation (direct oblimin). The Kaiser-Meyer-Oklin measure was .911, categorized as marvellous by Hutcheson and Sofroniou (1999). ). An initial analysis was run to obtain eigenvalues for each factor of the data. 4 factors had eigenvalues over Kaiser´s criterion of 1 and in combination explained 71% of the variance.

Table 2 shows the factor loadings after rotation.

The items that cluster on the same factor suggest that factor 1 represents positive outcome expectations, and factor 2 represents self-evaluative outcome expectations toward the level of physical activity. Factor 3 represents physical outcome expectations, and factor 4 represents negative self-evaluative outcome expectations.

Since the items loading on factor 1 include both physical and self-evaluative outcome expectations, this factor was retained.


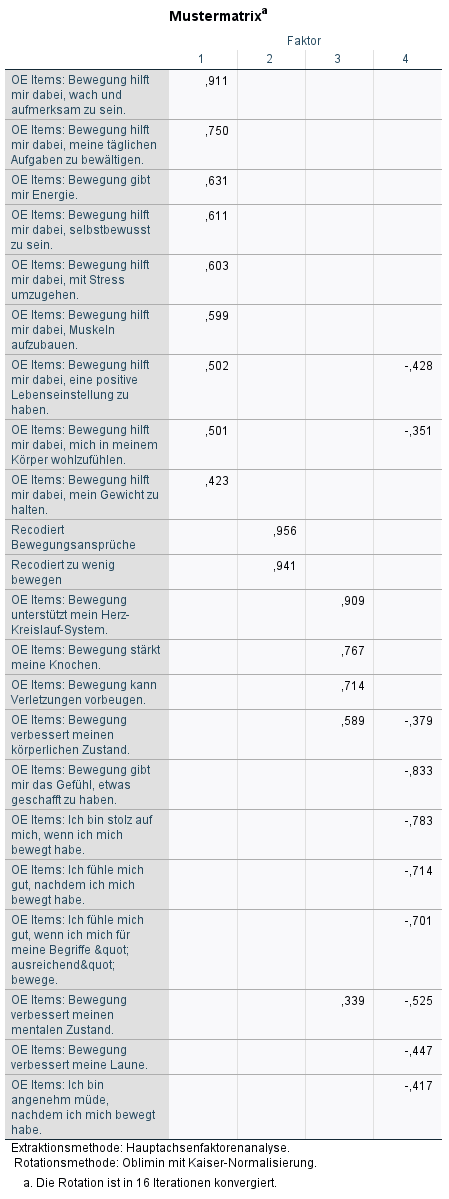


**Sociostructural Factors**

A principal axis factor analysis was conducted on the 7 items with oblique rotation (direct oblimin). The Kaiser-Meyer-Oklin measure was .792, categorized as middling by Hutcheson and Sofroniou (1999). One factor was extracted and explained 46% of the variance.

Table 3 shows the factor matrix.


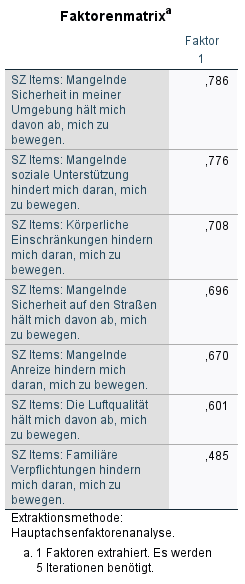


**Goals**

A principal axis factor analysis was conducted on the 7 items with oblique rotation (direct oblimin). The Kaiser-Meyer-Oklin measure was .826, categorized as meritorious by Hutcheson and Sofroniou (1999). An initial analysis was run to obtain eigenvalues for each factor of the data. 2 factors had eigenvalues over Kaiser´s criterion of 1 and in combination explained 68% of the variance.

Table 4 shows the factor loadings after rotation. The items that cluster on the same factor suggest that factor 1 represents the process of goal setting, whereas factor 2 represents the existence of concrete goals to be physically active.


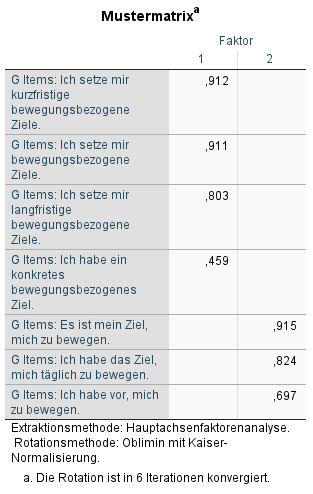

Supplement: Supplementary file 3 [file Datasheet3.docx]
