# Supplementary material for: Operationalization of the social cognitive theory to explain and predict physical activity in Germany: a scale development
Source: Front Sports Act Living. 2024 Nov 26;6:1508602. doi: 10.3389/fspor.2024.1508602 (PMC11628279; doi:10.3389/fspor.2024.1508602)
Supplement: Supplementary file 4 [file Datasheet4.docx]

Supplementary Material 4: ****Preliminary Factor Analysis****


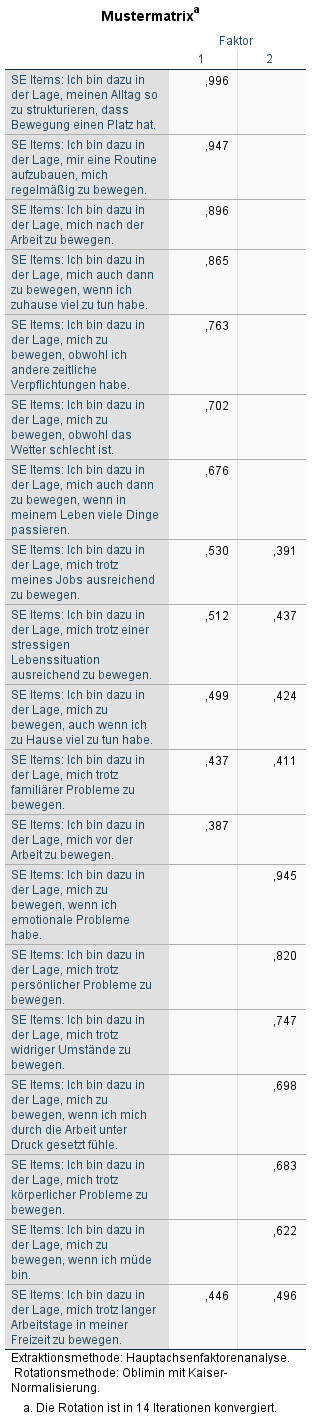


A principal axis factor analysis was conducted on the 28 items with oblique rotation (direct oblimin). The Kaiser-Meyer-Oklin measure was .866, categorized as meritorious by Hutcheson and Sofroniou (1999). An initial analysis was run to obtain eigenvalues for each factor of the data. 5 factors had eigenvalues over Kaiser´s criterion of 1 and in combination explained 66% of the variance.

Table 1 shows the factor loadings after rotation. The items that cluster on the same factor suggest that factor 1 represents self-efficacy, factor 2 represents sociostructural factors, factor 3 represents outcome expectations and factor 4 represents goals. Factor 5 represents another set of sociostructural factors.
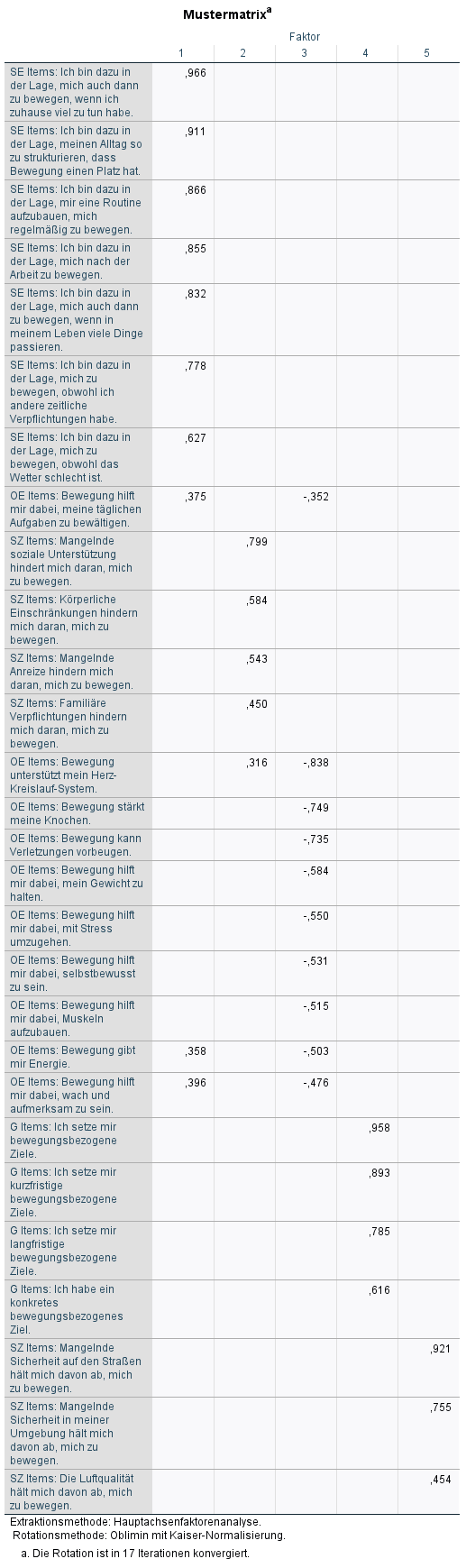


According to our retention criteria, the item pool was reduced to the items displayed in Table 2.

Table 2: Item pool after factor analysis

| Subscale | Items |
| --- | --- |
| Self-efficacy | 1. Ich bin dazu in der Lage, mich auch dann zu bewegen, wenn ich zuhause viel zu tun habe. 2. Ich bin dazu in der Lage, meinen Alltag so zu strukturieren, dass Bewegung einen Platz hat.^a^ 3. Ich bin dazu in der Lage, mir eine Routine aufzubauen, mich regelmäßig zu bewegen. 4. Ich bin dazu in der Lage, mich nach der Arbeit zu bewegen.^a^ 5. Ich bin dazu in der Lage, mich auch dann zu bewegen, wenn in meinem Leben viele Dinge passieren. 6. Ich bin dazu in der Lage, mich zu bewegen, obwohl ich andere zeitliche Verpflichtungen habe. 7. Ich bin dazu in der Lage, mich zu bewegen, obwohl das Wetter schlecht ist. |
| Sociostructural Factors | 1. Mangelnde soziale Unterstützung hindert mich daran, mich zu bewegen. 2. Körperliche Einschränkungen hindern mich daran, mich zu bewegen. 3. Mangelnde Anreize hindern mich daran, mich zu bewegen. 4. Familiäre Verpflichtungen hindern mich daran, mich zu bewegen. |
| Outcome Expectations | 1. Bewegung stärkt meine Knochen. 2. Bewegung kann Verletzungen vorbeugen. 3. Bewegung hilft mir dabei, mein Gewicht zu halten. 4. Bewegung hilft mir dabei, mit Stress umzugehen. 5. Bewegung hilft mir dabei, selbstbewusst zu sein. 6. Bewegung hilft mir dabei, Muskeln aufzubauen. ^a^ |
| Goals | 1. Ich setze mir bewegungsbezogene Ziele. 2. Ich setze mir kurzfristige bewegungsbezogene Ziele. 3. Ich setze mir langfristige bewegungsbezogene Ziele. 4. Ich habe ein konkretes bewegungsbezogenes Ziel. |

Note. ^a^ This item was removed from the item pool due to expert feedback as described in section 4 of the supplementary material.

Based on the feedback of the independent researcher, three additional items were excluded, two items from the preliminary self-efficacy scale and one item from the action outcome expectations. The respective items are marked accordingly in Table 2.
